# Ewing’s sarcoma of the sternum necessitating complex resection and reconstruction

**DOI:** 10.1080/23320885.2019.1598867

**Published:** 2019-04-15

**Authors:** Gino Vissers, Lucas Van Houtven, Jérôme Corthouts, Annemie Snoeckx, Marloes Luijks, Filip Thiessen, Thierry Tondu, Paul Van Schil

**Affiliations:** a Department of Plastic Surgery, Antwerp University Hospital, Edegem, Belgium;; b Faculty of Medicine and Health Sciences, Antwerp University, Wilrijk, Belgium;; c Department of Thoracic and Vascular Surgery, Sint Augustinus Hospital, Wilrijk, Belgium;; d Department of Radiology, Antwerp University Hospital, Edegem, Belgium;; e Department of Pathological Anatomy and Cytology, Sint Augustinus Hospital, Wilrijk, Belgium;; f Department of Thoracic and Vascular Surgery, Antwerp University Hospital, Edegem, Belgium

**Keywords:** Ewing’s sarcoma, sternal resection, sternal reconstruction, latissimus dorsi flap

## Abstract

We present a 30-year-old man with a sternal Ewing’s sarcoma, who was treated by complex resection of the sternal body and reconstruction by a methyl methacrylate sandwich graft and a pedicled latissimus dorsi flap.

## Introduction

Less than 1% of primary Ewing’s sarcoma occurs in the sternum [1]. The clinical presentation may consist of localised pain or swelling that develops in weeks or months. Most patients have subclinical metastases at presentation. A chest Computed Tomography (CT) scan and a Technetium 99m bone scintigraphy are recommended for detecting metastatic disease. The diagnosis is acquired by biopsy. Multimodality therapy including neo-adjuvant chemotherapy followed by surgery and/or radiotherapy has improved the 5-year survival rate up to 60–70% [2]. Surgery with radical resection is the preferred treatment. However, resection of the sternum and parts of the anterior chest wall imposes several clinical problems and reconstruction is mandatory to preserve its function.

## Case report

**Figure 1.**
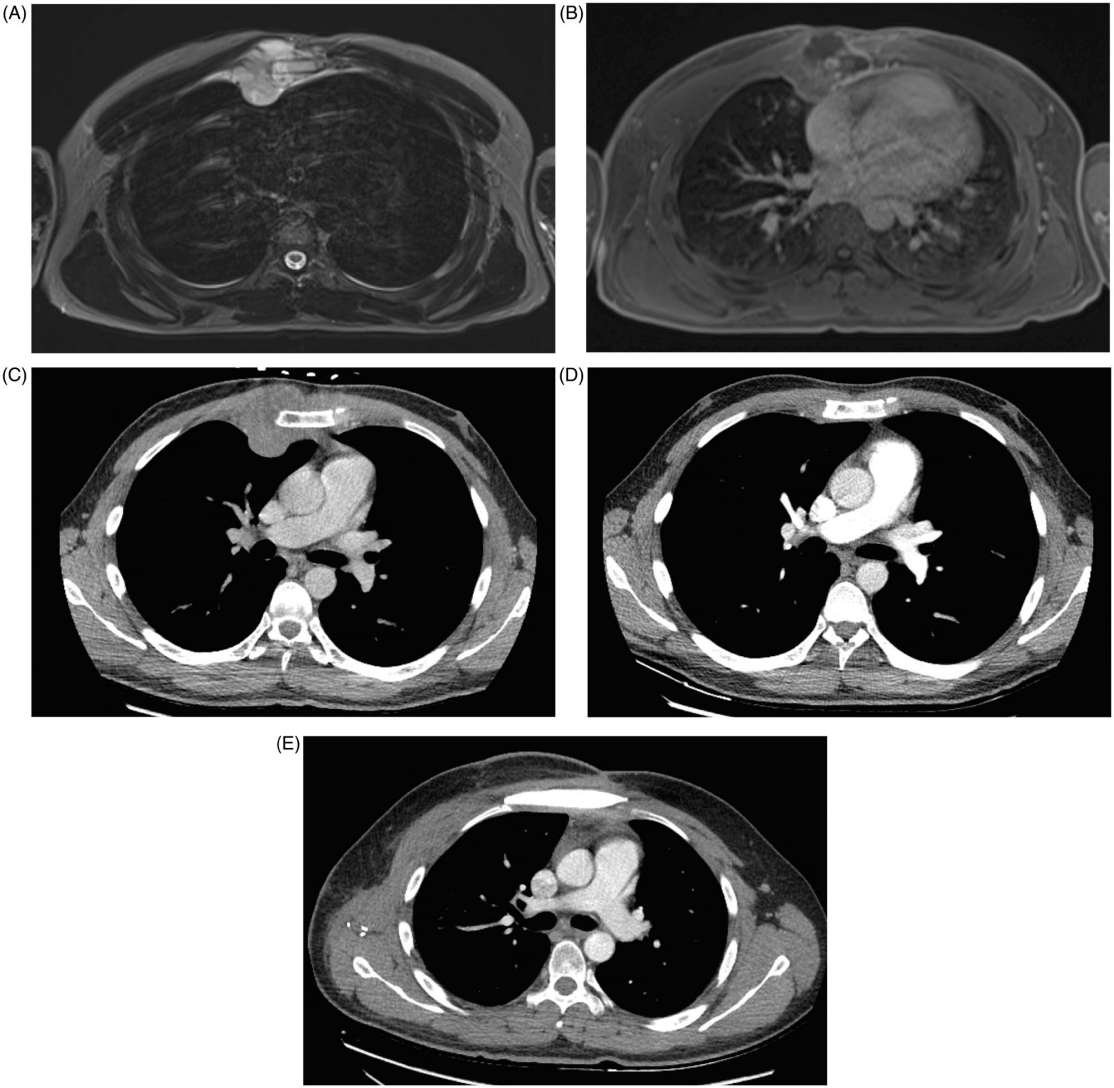
Axial T2-weighted MR-image (A) shows an area of abnormal signal intensity (bone involvement) in the sternum with associated right parasternal soft tissue component. The soft tissue mass has a lobulated morphology, is well defined and has a high signal intensity. Axial T1-weighted images after intravenous gadolinium contrast administration (B) show a heterogeneous lesion with peripheral enhancement and central areas of low signal intensity, corresponding to areas of necrosis. Axial CT-images at the time of diagnosis (C) show an abnormal density in the sternum with large soft tissue component. Follow-up CT after neoadjuvant chemotherapy (D) shows prominent shrinkage of the mass. Follow-up CT after surgery (E) shows normal postoperative findings after partial sternal resection and reconstruction.

**Figure 2.**
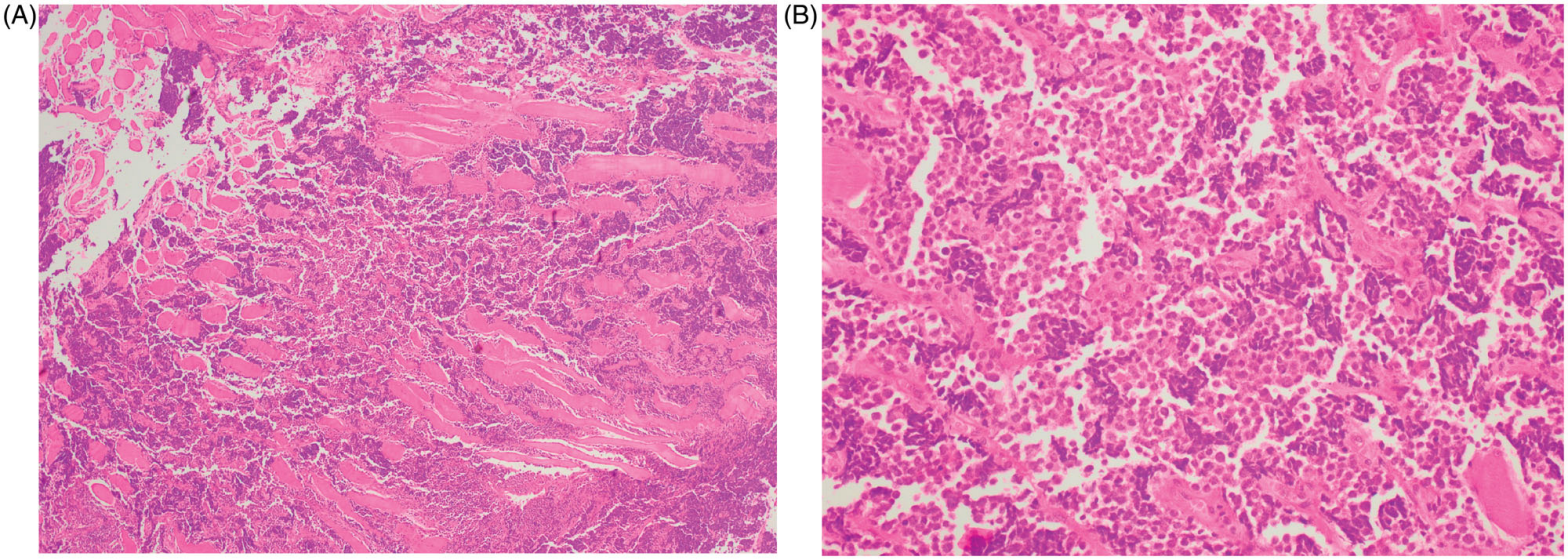
Sternal biopsy. HE stain (4× magnification) shows tumour cell infiltration between muscle fibres (A). HE stain (40× magnification) shows a high-grade population of ‘small blue round cells’, compatible with Ewing’s sarcoma (B).

A 30-year-old man was admitted at the emergency department with worsening chest pain for three weeks. Physical examination revealed a painful, fixed parasternal swelling at the fourth and fifth intercostal space. After initial assessment with ultrasound (not shown), CT and Magnetic Resonance Imaging (MRI) showed a parasternal mass with both an intraosseous and a large soft tissue component (Figure 1(A–C)). Positron Emission Tomography – Computed Tomography (PET-CT) and bone scan showed no distant metastases. Sternal biopsy revealed a high-grade population of ‘small blue round cells’, compatible with Ewing’s sarcoma (Figure 2).

The patient was treated with three series cycles of neo-adjuvant VIDE (Vincristine, Ifosfamide, Doxorubicin and Etoposide) chemotherapy with very good response on CT (Figure 1(D)). Subsequently, resection of the sternal body was performed via an elliptical parasternal incision incorporating the major pectoral muscles. Rib cartilages from the second till seventh rib were cut and both internal mammary arteries were sacrificed. Resection included the retrosternal mediastinal pleura and part of the right parietal pleura. The sternectomy was completed by a vertical osteotomy at the sternal angle. The resected sternal body measured 16 cm craniocaudal and 10 cm laterolateral. Histopathological examination of the sternal body showed a complete resection with negative margins but with >50% viable tumour cells (TNM 8th edition 2017: ypT1 N0(0/2) M0 R0).

**Figure 3.**
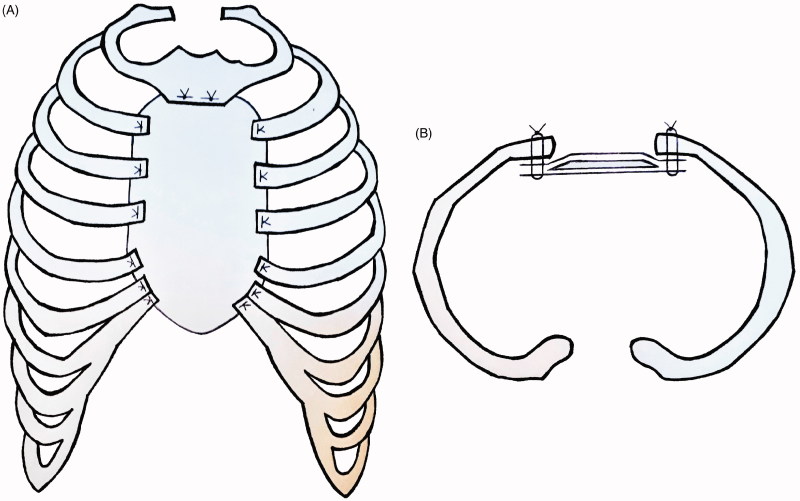
Cross section scheme of the sternal reconstruction with coronal plane (A), and axial plane (B). A layer of methyl methacrylate was sandwiched between two layers of a polypropylene mesh and sutured to the remaining ribs and muscles with polypropylene 3–0.

**Figure 4.**
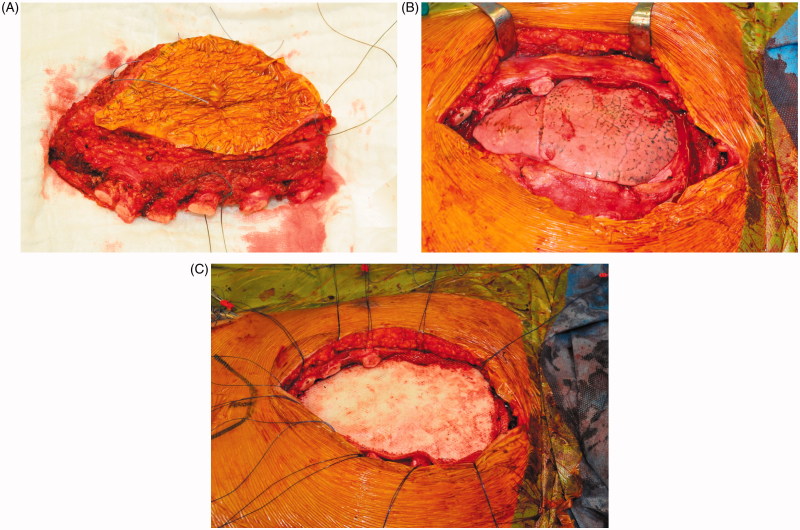
Intraoperative images of the resected sternal body incorporating the major pectoral muscles (A), the large defect of the anterior chest wall after resection (B), sternal reconstruction by a methyl methacrylate sandwich graft (C).

Sternal reconstruction was performed by a polypropylene – methyl methacrylate sandwich graft, which was sutured to the remaining ribs and muscles with polypropylene 3–0. Cross section schemas of the reconstruction are shown in Figure 3. The chest wall reconstruction was completed by a pedicled myocutaneous latissimus dorsi flap. The cutaneous part of the flap was designed as an ellipse, measuring the same dimensions as the sternal defect. The elliptical incision was made at the right posterior chest and dissection of the latissimus dorsi flap with the skin pedicle was carried out. The flap was transferred to the sternal defect through a subcutaneous pocket in the axillary fold. Closure in layers over surgical drains was performed. Intraoperative images are shown in Figure 4.

On the first postoperative day, the patient developed symptoms of dyspnoea, tachypnoea and increasing chest pain. Chest x-ray demonstrated a pneumothorax. This was a result of a cranial suture dehiscence of the flap. A pigtail catheter was inserted for chest drainage and the cranial defect was hermetically sealed after closure with extra staples. No further surgical complications were observed. The patient could leave the hospital in good general condition three weeks after surgery.

**Figure 5.**
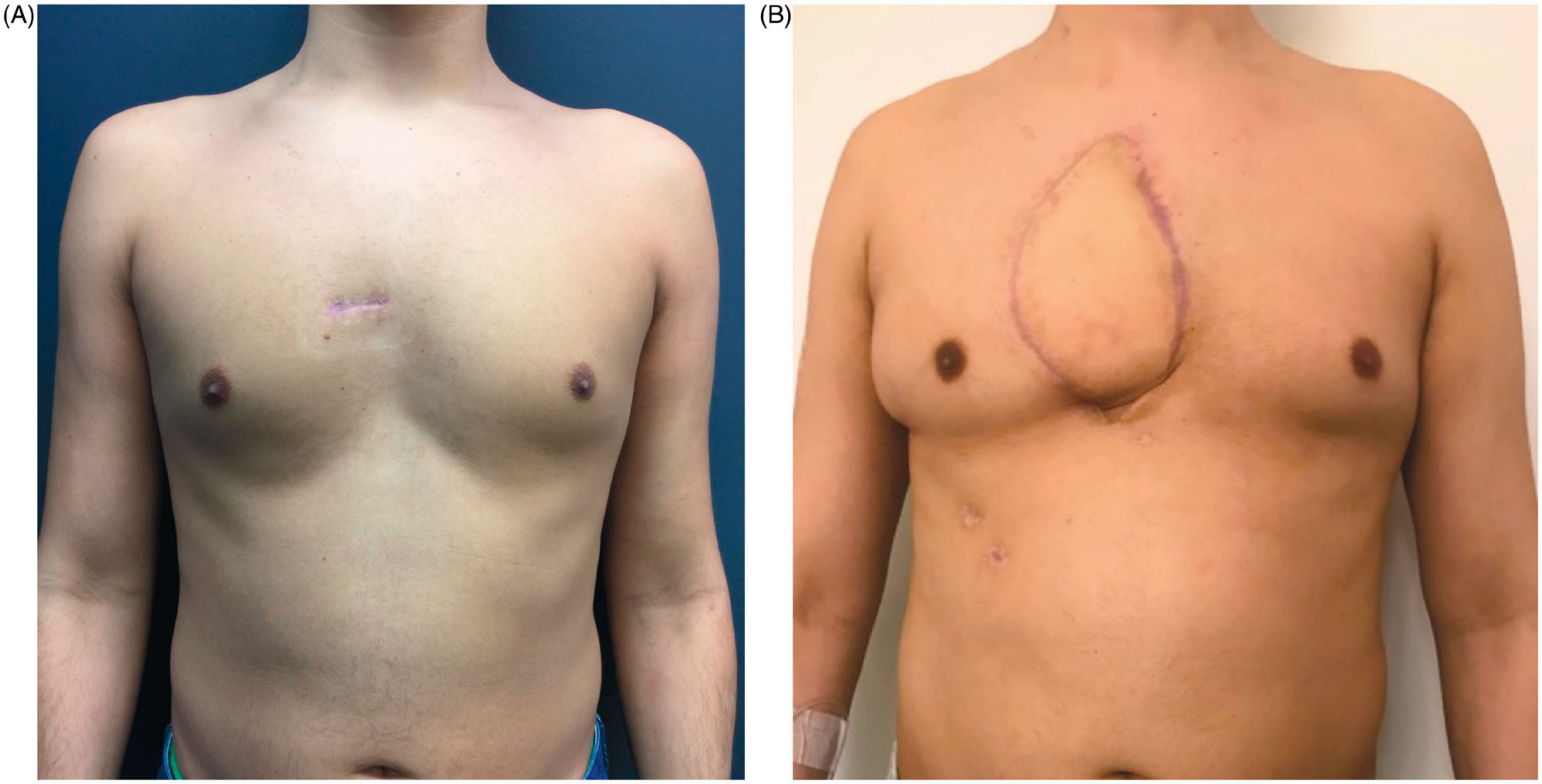
Comparison of the preoperative situation, which shows a small scar from the sternal biopsy at the presternal area (A), to the result four months after sternum resection and reconstruction by a polypropylene – methyl methacrylate sandwich graft and a pedicled myocutaneous latissimus dorsi flap (B).

A complete resection of a rare sternal tumour was accomplished, and a reliable reconstruction of a large precardiac defect with minimal donor site morbidities was obtained. The patient was seen in the outpatient department until discharge from clinic with no concerns four months after surgery. Figure 5 shows a comparison of the preoperative situation of the patient (Figure 5(A)) and the result four months postoperatively (Figure 5(B)).

## Discussion

Resection of the sternum and parts of the anterior chest wall with adequate negative margins is necessary to significantly improve survival. A wide resection according to the Enneking classification is achieved if tumour margins are at least 2 cm bilaterally [3]. Most centres incorporate healthy tissue margins of 3–5 cm to minimise local recurrence rates. This usually involves both the rib underneath and the rib above the affected level. During deeper dissection the pleura, thymus and lungs should be evaluated for possible tumour invasion and resected accordingly. Total sternectomy is not always necessary to achieve adequate margins, since subtotal or partial sternectomies can suffice.

Sternal reconstruction should consist of two steps: (1) Restoring chest wall stability, by suturing a mesh to adjacent ribs and fascia. The most commonly used meshes are synthetic non-absorbable patches (e.g. polypropylene, polytetrafluoroethylene (PTFE) and polyester). Polyglactin meshes are slowly absorbable and are believed to have lower infection rates due to their physiologic properties. These are similar to the more expensive biologic meshes, such as bovine pericardial prostheses that can be placed directly over the viscera. A methyl methacrylate substitute can be sandwiched between two layers of a mesh to improve the rigidity. Alternatively, titanium plates (such as the Ley prosthesis) can be used to support chest wall stability. Furthermore, sternal allograft and homograft transplantations become more commonly used, especially after oncological sternectomy or infective processes [4]. (2) Supplying soft tissue coverage. Direct suturing or skin grafts are often not sufficient to establish full tissue coverage. To cover the defect completely, advancement flaps (e.g. pectoralis major translation with skin advancement or breast flap), pedicled myocutaneous flaps (e.g. pectoralis major flap, latissimus dorsi flap, vertical or transverse rectus abdominis flap or omentum flap), pedicled perforator flaps [5], or free flaps (e.g. latissimus dorsi flap, deep inferior epigastric artery perforator flap) are valid options.

We chose the pedicled myocutaneous latissimus dorsi flap. It has the ability to fill a significant amount of intrathoracic space by passing between the ribs – after resection of the second and third rib to avoid vascular compression. It has a relatively short harvest time and it preserves the collateral blood supply to the sternum and parasternal tissues. Furthermore, the latissimus dorsi flap is not associated with an increased risk of hernia formation and has few donor site morbidities. Disadvantages of the flap are a possible insufficiency to cover the lower third of the sternum and the necessary repositioning to lateral position during surgery. Important risks and possible complications after a pedicled latissimus dorsi flap include hypertrophic scarring, wound problems, bleeding and haematoma formation, seroma formation, infection, numbness over the back, muscle weakness and postoperative chronic back pain.

When the internal mammary arteries are intact, a pedicled rectus abdominis flap would be a valid option too. Alternatively, reconstruction by an omentum flap has been described to reduce the risk of postoperative wound problems, especially after induction radiation therapy.

Although resection is the main treatment from a surgical point of view, it is important to realise that oncologic resection of sternal Ewing’s sarcoma is only part of a multimodality treatment. Subsequent reconstruction is often challenging as it requires both bone and soft tissue reconstruction of a large precardiac defect. Although several techniques exist, a combination of a methyl methacrylate sandwich graft with a pedicled myocutaneous latissimus dorsi flap is reliable with few donor site morbidities.

## References

[1] Paulussen M, Fr öhlich B, Jürgens H Ewing tumour: incidence, prognosis and treatment options. Paediatric Drugs. 2001;3:899–913. 11772151 10.2165/00128072-200103120-00003

[2] Bedetti B, Wiebe K, Ranft A, et al. Local control in Ewing sarcoma of the chest wall: results of the EURO-EWING 99 trial. Ann Surg Oncol. 2015;22:2853–2859. 26104542 10.1245/s10434-015-4630-0

[3] Widhe B, Bauer HC Surgical treatment is decisive for outcome in chondrosarcoma of the chest wall: a population-based Scandinavian Sarcoma Group study of 106 patients. J Thoracic Cardiovasc Surg. 2009;137:610–614. 10.1016/j.jtcvs.2008.07.024 19258076

[4] Sanna S, Brandolini J, Pardolesi A, et al. Materials and techniques in chest wall reconstruction: a review. J Vis Surg. 2017;3:95. 29078657 10.21037/jovs.2017.06.10 PMC5638032

[5] Hamdi M, Stillaert FB Pedicled perforator flaps in the trunk. Clin Plastic Surg. 2010;37:655–665. 10.1016/j.cps.2010.06.004 20816520

